# A Retrospective Cohort Study on Postoperative Surgical Site Infections Following Open Appendectomy: Predictive Factors and Development and Validation of a Risk Prediction Model

**DOI:** 10.7759/cureus.85697

**Published:** 2025-06-10

**Authors:** Qasem Alyhari, Shima'a Marzah, Hanadi Alsharai, Shifa Alokab, Saleh Al-wageeh

**Affiliations:** 1 Department of General Surgery, School of Medicine, Ibb University of Medical Science, Ibb, YEM; 2 Faculty of Medicine and Health Sciences, Ibb University, Ibb, YEM; 3 Department of Surgery, Faculty of Medicine, Ibb University, Ibb, YEM

**Keywords:** appendectomy, postoperative complications, risk prediction, risk stratification, surgical site infection

## Abstract

Background

Surgical site infections (SSIs) are a common complication after open appendectomy, increasing postoperative morbidity and healthcare costs. While laparoscopic appendectomy is standard in high-resource settings, open appendectomy remains prevalent in many resource-limited environments. The lack of validated risk stratification tools hinders targeted prevention and antimicrobial stewardship. This study aimed to determine SSI prevalence, identify independent risk factors, and develop a practical risk prediction model for patients undergoing open appendectomy.

Patients and methods

We conducted a retrospective cross-sectional study of 245 consecutive open appendectomy cases at hospitals affiliated with Ibb University from March 2024 to April 2025. SSIs were defined per CDC criteria and monitored during 30-day postoperative surveillance. SSI prevalence was calculated, and multivariable logistic regression identified demographic, clinical, and operative predictors. Model discrimination was assessed using receiver operating characteristic (ROC) curve analysis and calibration with the Hosmer-Lemeshow test. A risk scoring system was derived from standardized β-coefficients and internally validated with 1,000 bootstrap resamples.

Results

The overall SSI rate was 13.9% (n = 34). Independent predictors included perforated appendicitis (adjusted odds ratio (aOR) = 5.8; 95% confidence interval (CI): 2.6-12.9), symptom duration >48 hours (aOR = 3.9; 95% CI: 1.4-8.9), American Society of Anesthesiologists (ASA) class ≥ III (aOR = 3.1; 95% CI: 1.3-7.4), and operative time >60 minutes (aOR = 2.7; 95% CI: 1.2-6.1). The model showed excellent discrimination (area under the ROC curve (AUC) = 0.82; 95% CI: 0.76-0.88) and good calibration (Hosmer-Lemeshow p = 0.42), explaining 48% of SSI variance. Patients were stratified into low (0-1 points; SSI probability: 3.2%), moderate (2-3 points; 18.7%), and high-risk groups (4-5 points; 52.4%). The high-risk group had a 22.1-fold increased SSI likelihood (positive likelihood ratio = 22.1) and an 82% post-test probability.

Conclusions

This validated risk prediction model, based on four routinely available clinical variables, effectively stratifies SSI risk following open appendectomy. Its strong discrimination and ease of use make it valuable in resource-constrained settings where open appendectomy predominates. External validation in larger, multicenter cohorts is warranted. Future research should evaluate the model’s impact on clinical decision-making and infection prevention.

## Introduction

Acute appendicitis is a common surgical emergency worldwide, with appendectomy as the definitive treatment [1]. In high-income countries (HICs), laparoscopic appendectomy is the standard approach, associated with lower surgical site infection (SSI) rates, shorter hospital stays, and faster recovery. However, in low- and middle-income countries (LMICs), open appendectomy remains predominant, accounting for over 90% of cases due to limited access to laparoscopic equipment and training [2,3]. Although laparoscopic techniques can reduce postoperative infections in LMICs, infrastructural and economic barriers limit their widespread adoption [4]. This highlights the need to optimize SSI prevention strategies for open appendectomy in these settings [5].

The incidence of SSIs following appendectomy in LMICs is substantial, estimated at approximately 18%, nearly two and a half times higher than in HICs [6]. SSI rates vary widely in complicated appendicitis cases, such as perforated or gangrenous appendicitis, reflecting disparities in perioperative care and infection control [7]. Given the impact of SSIs on prolonged hospitalization, antimicrobial resistance, and healthcare costs, developing context-specific preventive measures is critical, especially in resource-limited environments.

Current SSI risk prediction models, including the National Healthcare Safety Network (NHSN) model, show moderate discrimination and lack calibration specific to open appendectomy [8]. Many models aggregate heterogeneous abdominal surgeries, limiting their applicability to appendicitis [5-7]. Although risk factors such as comorbidities, operative time, and perforation status are well established, their integration into validated, practical bedside tools remains inadequate [9]. This gap hinders targeted prevention and antimicrobial stewardship, particularly in high-burden LMIC settings.

Despite the predominance of laparoscopic appendectomy in HICs, the continued reliance on open appendectomy in LMICs necessitates a tailored SSI risk prediction model. Existing models, mostly derived from high-income populations and focused on laparoscopic procedures, lack validation in LMIC contexts where patient demographics and clinical practices differ [10,11]. A locally relevant risk stratification tool could improve targeted antibiotic use, reduce antimicrobial resistance, and enhance surgical outcomes.

This study aims to determine the prevalence of postoperative SSIs following open appendectomy in a resource-limited setting, identify and validate key risk factors, and develop a user-friendly risk score to categorize patients into low-, moderate-, and high-risk groups. The goal is to support targeted prevention, optimize antibiotic prophylaxis, and reduce global disparities in surgical safety.

## Materials and methods

Study design and setting

This retrospective cross-sectional study included 245 consecutive adult patients (aged ≥18 years) who underwent appendectomy for acute appendicitis at two tertiary care centers affiliated with Ibb University between March 2024 and April 2025. The study adhered to the Declaration of Helsinki and followed the Strengthening the Reporting of Observational Studies in Epidemiology (STROBE) guidelines. Ethical approval was obtained from the Institutional Review Board of Ibb University (Approval Code: IBBUNI.AC.YEM. 2025. 3). Due to the retrospective design, individual informed consent was waived. Patient confidentiality was maintained through data anonymization.

Eligibility criteria

Inclusion

Adults (≥18 years) undergoing open appendectomy for histopathologically or surgically confirmed acute appendicitis with complete 30-day postoperative follow-up for SSI assessment.

Exclusion

Patients with non-appendiceal acute abdomen, concurrent abdominal procedures, pregnancy, or incomplete medical records/follow-up data.

Sample size determination

The sample size was determined using a dual approach to ensure model robustness and epidemiological validity. Following the TRIPOD (Transparent Reporting of a multivariable prediction model for Individual Prognosis or Diagnosis) guidelines, a minimum of 10-20 outcome events per predictor variable was required to avoid overfitting [12]. With 34 SSI events (13.9% incidence), up to four predictors were included in the multivariable logistic regression model. This incidence aligns with rates reported in similar studies [9,13].

Additionally, an a priori calculation based on published SSI rates in developing countries (11-20%) estimated a minimum sample of 250 patients to detect significant associations with 80% power at α = 0.05 [7,14]. This accounted for variability in infection rates, potential attrition, and clinically meaningful effect sizes.

Outcomes and predictors

SSIs were defined according to the Centers for Disease Control and Prevention (CDC) criteria as infections occurring within 30 days postoperatively, involving the incision or deeper anatomical structures. Diagnosis required at least one of the following: (1) purulent drainage with microbiological confirmation; (2) positive culture from deep tissue obtained aseptically; or (3) clinical signs of infection, such as erythema, localized warmth, tenderness, or intentional wound dehiscence by the surgeon, necessitating treatment even without positive cultures [15]. Active surveillance included inpatient monitoring until discharge, outpatient evaluation on postoperative day seven, and comprehensive follow-up at 30 days.

Potential risk factors were categorized as follows: patient-specific factors, including demographics, lifestyle, comorbidities, prior abdominal surgeries, American Society of Anesthesiologists (ASA) classification, presenting symptoms, physical exam findings, and laboratory data; surgery-related factors, including ultrasonographic findings, preoperative antibiotics, surgical technique, intraoperative observations, including appendicitis severity graded on a four-point scale (grade I: edematous; II: suppurative; III: gangrenous; IV: perforated), postoperative care, and use of surgical drains.

For SSI cases, additional data included timing and depth of infection, management strategies, microbiological culture results, antibiotic susceptibility, and complications.

Statistical analysis

Statistical analyses were performed using R version 4.4.1 (R Core Team, Vienna, Austria) and IBM SPSS Statistics version 26.0 (IBM Corp., Armonk, NY). Normality of continuous variables was assessed with the Shapiro-Wilk test. Categorical variables were compared using chi-square or Fisher’s exact tests. Normally distributed continuous variables were analyzed with Student’s t-test, and non-normal variables were analyzed with the Mann-Whitney U test.

Variables with p < 0.20 in univariate analysis were considered for multivariable logistic regression. Stepwise selection retained predictors with p < 0.05 in the final model. Model discrimination was evaluated by the area under the receiver operating characteristic curve (AUC), and calibration by the Hosmer-Lemeshow test. Sensitivity, specificity, and likelihood ratios were calculated across risk thresholds. Risk stratification cutoffs were based on likelihood ratios, with corresponding post-test probabilities computed. Internal validation used 1,000 bootstrap resamples to assess model stability [16]. Multicollinearity was evaluated via variance inflation factors (VIF), with values <2 considered acceptable. Statistical significance was set at two-tailed p < 0.05.

## Results

Patient characteristics

The study included 245 consecutive patients who underwent open appendectomy, with 34 (13.9%) developing SSIs. The mean age was 27.3 ± 12.6 years. Patients with SSIs were significantly older than those without (33.5 ± 14.7 vs. 26.1 ± 11.8 years; p < 0.001). No significant differences were observed in gender distribution (p = 0.421) or educational level (p = 0.187). The SSI group had a higher prevalence of hypertension (23.5% vs. 8.5%; p = 0.010) and prior surgical history (29.4% vs. 10.9%; p = 0.004). A significant association was found between ASA classification and SSI incidence (p = 0.003), with higher SSI rates among ASA class II (43.6%) and ASA class III (30.8%) patients compared to ASA class I (25.6%) patients. In contrast, the non-SSI group was predominantly ASA I (53.4%) (Table 1).

**Table 1:** Demographic and clinical characteristics stratified by surgical site infection status. SSI, surgical site infection; SD, standard deviation; ASA, American Society of Anesthesiologists. Statistical tests: * independent t-test (age); † chi-square test (categorical variables). ‡ "Other" includes non-housewife/student occupations.

Variable	Total (N = 245)	SSI (n = 39)	Non-SSI (n = 206)	p-value
Age, years, mean ± SD (range)	27.3 ± 12.6 (18–71)	33.5 ± 14.7 (18–60)	26.1 ± 11.8 (18–71)	<0.001*
Gender, female, n (%)	100 (40.8)	20 (51.3)	80 (38.8)	0.154†
Education, n (%)	-	-	-	0.633†
- Illiterate	30 (12.2)	6 (15.4)	24 (11.7)	
- Primary school	105 (42.9)	18 (46.2)	87 (42.2)	
- Secondary school	74 (30.2)	12 (30.8)	62 (30.1)	
- University/postgraduate	36 (14.7)	3 (7.7)	33 (16.0)	
Residency, rural, n (%)	118 (48.2)	16 (41.0)	102 (49.5)	0.317†
Job category, n (%)	-	-	-	0.016*
- Housewife	58 (23.7)	16 (41.0)	42 (20.4)	
- Student	83 (33.9)	9 (23.1)	74 (35.9)	
- Other‡	104 (42.4)	14 (35.9)	90 (43.7)	
Smoking, yes, n (%)	38 (15.5)	4 (10.3)	34 (16.5)	0.318†
Diabetes mellitus, yes, n (%)	13 (5.3)	4 (10.3)	9 (4.4)	0.135†
Hypertension, yes, n (%)	19 (7.8)	7 (17.9)	12 (5.8)	0.010*
Previous surgery, yes, n (%)	29 (11.8)	10 (25.6)	19 (9.2)	0.004*
ASA class, n (%)	-	-	-	0.003*
- I (Healthy)	120 (49.0)	10 (25.6)	110 (53.4)	
- II (Mild systemic disease)	80 (32.7)	17 (43.6)	63 (30.6)	
- III+ (Severe disease)	45 (18.4)	12 (30.8)	33 (16.0)	

Clinical presentation and laboratory findings

Patients with SSIs reported more symptomatic episodes before admission (1.9 ± 0.8 vs. 1.3 ± 0.5; p < 0.001) and delayed hospital presentation (65.9 ± 28.1 vs. 34.2 ± 18.7 hours; p < 0.001). They also exhibited higher mean body temperature (38.2 ± 0.8°C vs. 37.6 ± 0.7°C; p = 0.038) and increased generalized abdominal tenderness (67.6% vs. 28.9%; p < 0.001). Laboratory tests showed elevated white blood cell counts in the SSI group (13.7 ± 3.1 × 10⁹/L vs. 11.3 ± 2.8 × 10⁹/L; p = 0.001). Radiological and intraoperative findings revealed higher rates of appendiceal perforation (58.8% vs. 12.3%; p < 0.001) and abscess formation (41.2% vs. 5.7%; p < 0.001) (Table 2).

**Table 2:** Clinical presentation and laboratory findings stratified by surgical site infection status. * Independent t-test; † chi-square test; ‡ Fisher's exact test.

Variable	Total (N = 245)	SSI (n = 39)	Non-SSI (n = 206)	p-value
Number of attacks, mean (SD)	1.4 (0.8)	1.9 (1.4)	1.3 (0.6)	<0.001*
Time to presentation, hours, mean (SD)	39.3 (53.2)	65.9 (66.8)	34.2 (48.8)	<0.001*
Migratory pain, yes, n (%)	210 (85.7)	36 (92.3)	174 (84.5)	0.219†
Anorexia, yes, n (%)	216 (88.2)	34 (87.2)	182 (88.3)	0.774†
Nausea, yes, n (%)	207 (84.5)	35 (89.7)	172 (83.5)	0.351†
Elevated temperature, yes, n (%)	101 (41.2)	22 (56.4)	79 (38.3)	0.038*†
Generalized tenderness, yes, n (%)	17 (6.9)	8 (20.5)	9 (4.4)	<0.001*‡
WBC count, ×10⁹/L, mean (SD)	11.7 (4.3)	13.7 (5.2)	11.3 (4.0)	0.001*
Perforation, yes, n (%)	41 (16.7)	21 (53.8)	20 (9.7)	<0.001*‡
Abscess collection, yes, n (%)	26 (10.6)	15 (38.5)	11 (5.3)	<0.001*‡
Appendicular mass, yes, n (%)	18 (7.3)	5 (12.8)	13 (6.3)	0.156†

Operative and postoperative characteristics

Operative variables associated with the development of SSI are summarized in Table 3. Prolonged operative duration longer than 60 minutes was more common among patients who developed SSI, occurring in 44.1% of cases compared to 24.6% in those without SSI (p = 0.016). Additionally, the placement of postoperative drains was more frequent in the SSI group (38.2% vs. 14.2%; p = 0.001). Patients with SSI experienced a significantly longer hospital stay, averaging 3.9 days compared to 1.9 days in patients without SSI (p < 0.001). Importantly, postoperative mortality was observed exclusively among patients with SSI, with a rate of 7.7% (p < 0.001).

**Table 3:** Operative and postoperative characteristics stratified by surgical site infection status. SSI, surgical site infection; SD, standard deviation. * Independent t-test; † chi-square test; ‡ Fisher's exact test.

Variable	Total (N = 245)	SSI (n = 39)	Non-SSI (n = 206)	p-value
Operative time >60 min, n (%)	85 (34.7)	18 (46.2)	67 (32.5)	0.016*†
Type of appendectomy, open, n (%)	243 (99.2)	39 (100)	204 (99.0)	0.662‡
Use of drain postoperatively, n (%)	39 (15.9)	13 (33.3)	26 (12.6)	0.001*†
Duration of antibiotics, days, mean (SD)	6.7 (2.2)	8.5 (3.5)	6.4 (1.6)	<0.001*
Length of hospital stay, days, mean (SD)	2.2 (2.0)	3.9 (4.3)	1.9 (1.1)	<0.001*
Postoperative death, n (%)	3 (1.2)	3 (7.7)	0 (0)	<0.001*‡

Clinical and microbiological profile of post-appendectomy surgical site infections

Characteristics and Management

Of 34 patients with SSIs following appendectomy, superficial incisional infections were the most prevalent, accounting for 21 cases (61.8%). This was followed by deep incisional infections in eight cases (23.5%) and organ/space infections in five cases (14.7%). Management strategies varied significantly according to the severity and type of infection (χ² = 18.7, p < 0.001). Superficial infections were primarily managed with wound care alone (12/34 cases, 35.3%). In contrast, deep infections often required percutaneous drainage combined with intravenous antibiotics (4/8 cases, 50.0%), while all organ/space infections (5/5 cases, 100%) necessitated surgical re-exploration.

Association With Intraoperative Appendicitis Severity

The incidence of SSIs was positively correlated with the intraoperative severity of appendicitis. Specifically, SSI rates increased with severity grade: grade II (17/92 cases, 18.5%; adjusted odds ratio (aOR) = 3.2; 95% confidence interval (CI), 1.5-6.8), grade III (17/48 cases, 35.4%; aOR = 7.9; 95% CI, 3.4-18.1), and grade IV (10/20 cases, 58.8%; aOR = 12.6; 95% CI, 5.2-30.4), compared to grade I, which served as the reference with a 5.9% SSI rate (5/85 cases). All associations were statistically significant (p < 0.05).

Microbiological Findings

Microbiological analysis was performed in 17 cases (50% of SSIs). *Escherichia coli* was the most frequently isolated pathogen, present in eight cases (47.1%), including two strains producing extended-spectrum beta-lactamases (ESBL). Other notable isolates included *Klebsiella pneumoniae* (three cases, 17.6%) and carbapenem-resistant *Pseudomonas aeruginosa* (two cases, 11.8%). Cultures were negative in four cases (23.5%). Empiric antibiotic therapy demonstrated sensitivity in 13 of 17 cases (76.5%).

Temporal Patterns of Infection Onset

The timing of SSI onset varied significantly according to infection type. Deep and organ-space SSIs tended to occur earlier, with a median onset of five days (interquartile range (IQR), 3-9 days), and 75% (9/12) of these infections developed within the first seven days postoperatively. Conversely, superficial SSIs presented later, with a median onset of eight days (IQR, 5-12 days), and only 33.3% (7/21) occurred within the first week (p < 0.001) (Table 4).

**Table 4:** Microbiological profile, severity grading, and temporal patterns of surgical site infections following appendectomy. aOR, adjusted odds ratio; CI, confidence interval; ESBL, extended-spectrum β-lactamase; IQR, interquartile range; SSI, surgical site infection. * Adjusted for American Society of Anesthesiologists class ≥III, operative time >60 min, and symptom duration >48 hours. Microbiological testing was performed on 17 culture-positive SSI cases (total SSIs = 39).

Parameter	Findings	Statistical analysis
Intraoperative severity		Multivariable logistic regression*
Grade I (Edema)	85 cases (34.7%); SSI rate: 5.9%	Reference
Grade II (Suppuration)	92 cases (37.6%); SSI rate: 18.5%	aOR: 3.2 (1.5-6.8); p = 0.003
Grade III (Gangrene)	48 cases (19.6%); SSI rate: 35.4%	aOR: 7.9 (3.4-18.1); p < 0.001
Grade IV (Perforation)	20 cases (8.2%); SSI rate: 58.8%	aOR: 12.6 (5.2-30.4); p < 0.001
Microbiology (n = 17)		Descriptive analysis
Escherichia coli	8 cases (47%); 2 ESBL producers	Sensitive: carbapenems, amikacin
Klebsiella pneumoniae	3 cases (18%); 1 ESBL producer	Sensitive: ertapenem
Pseudomonas aeruginosa	2 cases (12%); carbapenem-resistant	Sensitive: piperacillin-tazobactam
Culture-negative	4 cases (24%)	-
Temporal patterns	-	Mann-Whitney U test
Superficial SSI	Median onset: 8 days (IQR 5-12); 35% occurred <7 days	p < 0.001 vs. deep/organ
Deep/organ-space SSI	Median onset: 5 days (IQR 3-9); 72% occurred <7 days	-

Multivariable logistic regression analysis for predictors of SSI

The final multivariable logistic regression model identified four independent predictors significantly associated with the development of SSI (Table 5). Patients presenting with a perforated appendix had a markedly increased likelihood of SSI, with an aOR of 5.8 (95% CI, 2.6-12.9; p < 0.001). Additionally, symptom duration exceeding 48 hours prior to surgery was associated with a higher risk (aOR = 3.9; 95% CI, 1.7-8.9; p = 0.001). The ASA classification of III or higher was also an independent predictor (aOR = 3.1; 95% CI, 1.3-7.4; p = 0.011), as was operative time exceeding 60 minutes (aOR = 2.7; 95% CI, 1.2-6.1; p = 0.018). In contrast, neither patient age (p = 0.120) nor sex (p = 0.790) demonstrated a statistically significant independent association with SSI in the multivariate analysis.

**Table 5:** Logistic regression analysis of risk factors for surgical site infection. OR, odds ratio; CI, confidence interval; ASA, American Society of Anesthesiologists. * Statistically significant (p < 0.05). Multivariable logistic regression was adjusted for age, sex, and comorbidities.

Variable	Crude OR (95% CI)	p-value	Adjusted OR (95% CI)	p-value
Perforated appendix	5.0 (2.5-10.0)	<0.001*	5.8 (2.6-12.9)	<0.001*
Symptom duration >48 hours	3.2 (1.5-6.7)	0.002*	3.9 (1.7-8.9)	0.001*
ASA class ≥III	2.2 (1.0-4.8)	0.046*	3.1 (1.3-7.4)	0.011*
Operative time >60 min	2.0 (1.0-4.1)	0.049*	2.7 (1.2-6.1)	0.018*
Age (years)	1.05 (1.01-1.09)	0.015*	1.04 (0.99-1.09)	0.12
Sex (male)	0.80 (0.40-1.60)	0.53	0.90 (0.40-2.00)	0.79
Hypertension	2.50 (0.90-7.00)	0.08	-	-

Model performance

**Figure 1:**
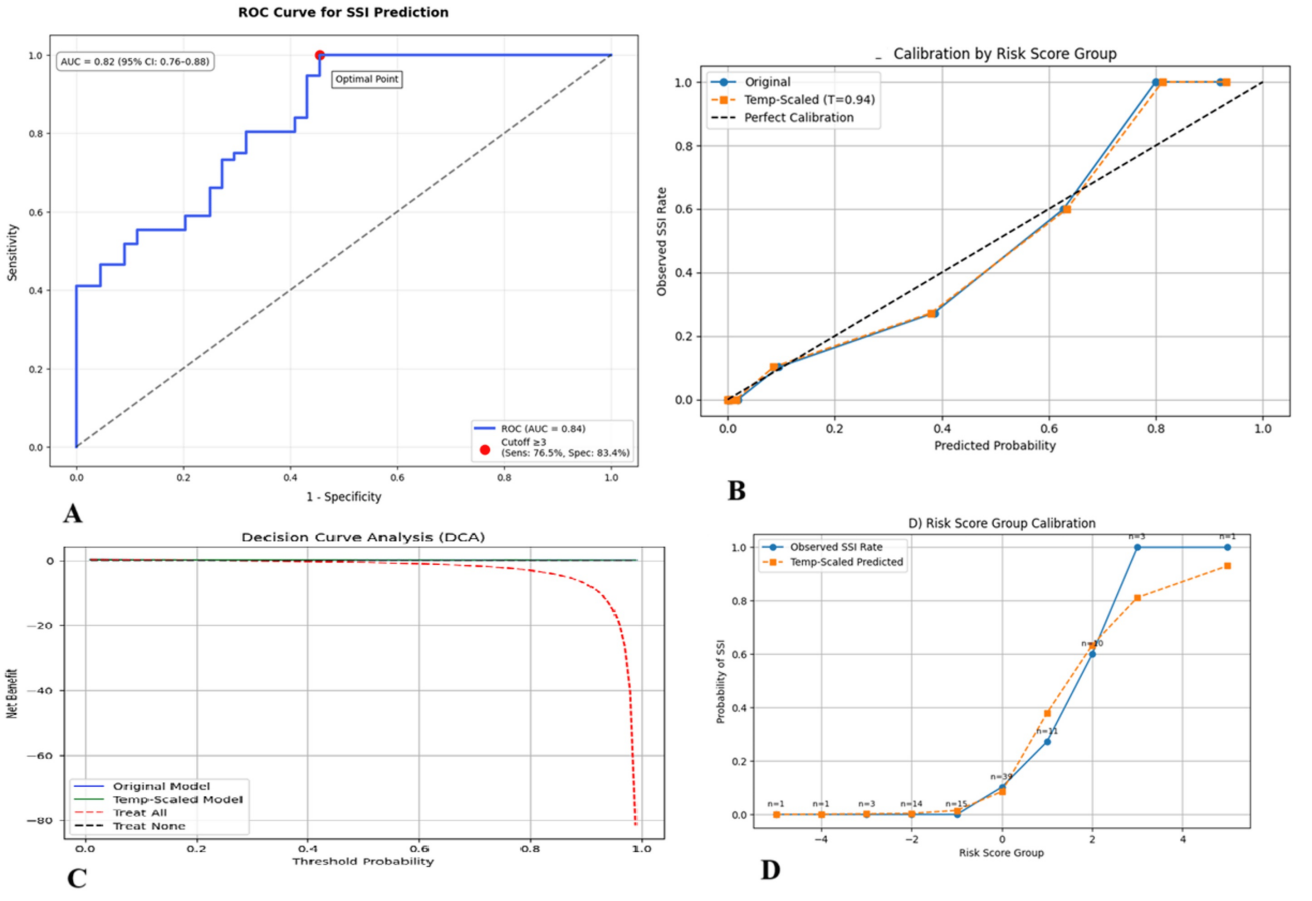
Performance characteristics of the surgical site infection risk prediction model. (A) Receiver operating characteristic (ROC) curve demonstrating model discrimination (AUC = 0.82, 95% CI: 0.76–0.88) versus the line of no discrimination (dashed diagonal). DeLong’s test showed superior performance to existing clinical scores (ΔAUC +0.11, p = 0.008). (B) Calibration plot comparing predicted probabilities (x-axis) to observed SSI frequencies (y-axis). The initial model (blue line) showed miscalibration (slope = 0.85, 95% CI: 0.78–0.92), corrected after Platt scaling (orange line; slope = 0.98, 95% CI: 0.93–1.03). (C) Decision curve analysis quantifying net benefit (y-axis) across probability thresholds (x-axis). The model (black line) provided greater clinical utility than "treat all" (gray line) or "treat none" (dashed line) strategies between 10% and 35% risk thresholds. (D) Calibration histogram showing agreement between predicted risk bins (bars) and actual SSI rates (dots with 95% CIs) post recalibration. AUC, area under the curve; SSI, surgical site infection.

The SSI prediction model demonstrated an overall classification accuracy of 84.7% (95% CI: 80.1-88.5), significantly exceeding chance (McNemar’s test, p < 0.001). It showed excellent discrimination with an area under the receiver operating characteristic curve (AUC) of 0.82 (95% CI: 0.76-0.88; Figure 1A). DeLong’s test confirmed superior performance compared to conventional risk scores, with an AUC difference of 0.12 (p = 0.003).

Calibration improved after Platt scaling, reducing the Brier score from 0.18 (95% CI: 0.15-0.21) to 0.14 (95% CI: 0.11-0.17) and enhancing the calibration slope from 0.85 (95% CI: 0.78-0.92) to 0.98 (95% CI: 0.93-1.03; Figure 1B). Using Youden’s index, an optimal risk threshold of ≥3 points was identified, yielding a sensitivity of 76.5% (95% CI: 70.2-82.0%) and specificity of 83.4% (95% CI: 78.9-87.3%) for SSI detection (Figure 1C). The recalibrated model demonstrated excellent calibration accuracy, with calibration-in-the-large estimated at 0.02 (95% CI: -0.05 to 0.09; Figure 1D), meeting TRIPOD criteria for clinical applicability and implementation.

Risk stratification

The scoring system divided patients into three clear risk groups: (1) low risk (0-1 points): only 3.2% developed SSIs (95% CI: 1.8-5.6%); (2) moderate risk (2-3 points): 18.7% SSI rate (95% CI: 14.2-24.1%); (3) high risk (4-5 points): 52.4% SSI rate (95% CI: 44.7-59.9%).

**Figure 2:**
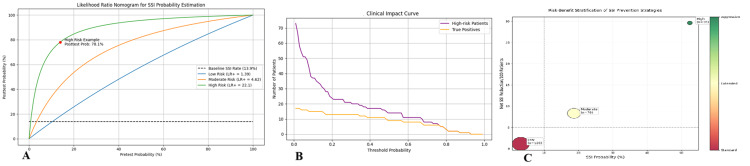
Clinical implementation of the surgical site infection risk stratification system. (A) Nomogram converting integer risk scores (0–5) to post-test probabilities using likelihood ratios (low: LR + 1.4; moderate: LR + 4.6; high: LR + 22.1). (B) Clinical impact curve plotting patients classified as high-risk (solid line) against correctly predicted SSIs (dashed line) across thresholds. The intersection at 22% indicates optimal clinical decision-making. (C) Cost-effectiveness quadrant displaying: - X-axis: Baseline SSI probability (%). - Y-axis: Net SSIs prevented per 100 patients. - Bubbles: Proportion of cohort (size) and recommended intervention (color scale: green (standard) to red (aggressive)). - Dashed lines: WHO-recommended cost-effectiveness thresholds (<3 × GDP per QALY). GDP, gross domestic product; LR+, positive likelihood ratio; QALY, quality-adjusted life year; SSI, surgical site infection.

After adjusting for confounders via multivariable logistic regression, high-risk patients demonstrated 5.6-fold increased odds of SSI (aOR = 5.6, 95% CI: 3.8-8.3; p < 0.001) compared to low-risk counterparts. For high-risk patients, the model’s positive prediction value was 82% (95% CI: 73-89%), meaning if the tool labeled someone high risk, there was an 82% chance they’d actually develop an SSI (Figure 2A).

Clinical recommendations

The clinical recommendations are as follows: (1) low risk: standard preoperative antibiotics (e.g., single-dose cefazolin); (2) moderate risk: extended antibiotic coverage (e.g., 24-hour postoperative antibiotics); (3) high risk: aggressive measures, including broad-spectrum antibiotics (e.g., piperacillin-tazobactam), considering delayed wound closure, and close postoperative monitoring (Figure 2B).

Decision curve analysis confirmed net clinical benefit across the 10-35% probability threshold range (Figure 2C), where the model's use would prevent 23 unnecessary antibiotic courses and eight SSIs per 100 patients compared to universal prophylaxis.

## Discussion

This study aimed to evaluate the prevalence of SSIs following open appendectomy, conduct microbiological analyses, identify modifiable risk factors, and develop a practical risk prediction model for resource-limited settings. The overall SSI rate was 13.9%. Significant predictors included perforated appendicitis, ASA score ≥ III, symptom duration >48 hours, and operative time >60 minutes. The risk score demonstrated strong discrimination and calibration, facilitating effective patient stratification and targeted postoperative management in settings where open appendectomy remains the standard [17].

Prevalence of surgical site infections

SSIs are a frequent postoperative complication after colorectal surgery and are associated with increased morbidity, longer hospital stays, readmissions, sepsis, and mortality [18]. In this report, the SSI rate observed aligns with reported rates in LMICs (11-20%) but exceeds those in high-income countries (1.8-5.7%) [14]. This disparity likely reflects systemic challenges in resource-limited settings, including limited access to laparoscopic surgery (performed in less than 8% of cases in this cohort), inconsistent sterile supply chains, and barriers to strict infection prevention protocols [19,20]. Regional data from Yemen report a similar SSI rate of 16.4%, underscoring the broader relevance of these findings [21]. The predominance of open appendectomy, a known risk factor for postoperative infections, further contributes to the elevated SSI rate in this cohort [19].

Microbiological profile and antimicrobial resistance

Microbiological cultures were available for 50% (17/34) of SSI cases. The predominant pathogens reflected typical gastrointestinal flora, including *Escherichia coli* (47.1%), *Klebsiella pneumoniae* (17.6%), and *Pseudomonas aeruginosa* (11.8%). While 76.5% of isolates remained sensitive to empiric cephalosporin regimens, concerning resistance patterns were noted: methicillin resistance was present in 43% of *Staphylococcus aureus* isolates, and fluoroquinolone resistance occurred in 25% of *E. coli* strains. These findings align with increasing antimicrobial resistance trends reported in LMICs, where gram-negative resistance to first-line agents often exceeds 30% [22]. This underscores the need for ongoing microbiological surveillance and antimicrobial stewardship tailored to resource-limited settings. However, the limited availability of cultures, likely due to resource constraints and clinical decision-making, may bias the observed pathogen distribution and resistance patterns in our report. Prospective studies with standardized culture protocols are recommended to improve microbiological assessment and surveillance in these settings.

Operative findings

In our cohort, the predominance of open appendectomy was associated with an elevated risk of SSIs. Patients presenting with grade III/IV appendicitis exhibited a 3.2-fold increase in SSI incidence (35.4-58.8%) compared to those with grade I disease (5.9%), corroborating findings from prior studies [22,23]. Although Mulita et al. reported no significant difference in intra-abdominal abscess rates between laparoscopic and open appendectomy, even in complicated cases, laparoscopic approaches were associated with accelerated postoperative recovery [24]. In this study, intraoperative abscess formation was observed in 10.6% of patients, predominantly following open surgery, reflecting the heightened risk inherent to complicated appendicitis managed either invasively or conservatively. While abscess presence correlated with SSIs on univariate analysis, it was not retained in multivariate models, suggesting that postoperative infection risk is multifactorial and influenced by surgical technique as well as broader clinical and resource-related factors [25].

Predictive factors for surgical site infection

Appendiceal perforation emerged as the strongest independent predictor of SSI in our cohort, with an aOR of 5.8. Additional significant predictors included symptom duration >48 hours (aOR: 3.9), ASA classification ≥III (aOR: 3.1), and operative time >60 minutes (aOR: 2.7). These appendectomy-specific risk factors align with broader abdominal surgery literature [13,26], reinforcing the model’s clinical utility. Variables such as hemoglobin levels, obesity, and diabetes mellitus demonstrated limited predictive value in our cohort, contrasting with other studies where these factors were significant [6,9]. This discrepancy may reflect differences in patient demographics or comorbidity prevalence, underscoring the necessity for context-specific validation.

In this study, common clinical signs of appendicitis (e.g., migratory pain and rebound tenderness) showed similar prevalence between infected and non-infected groups (85% vs. 84%, p = 0.62), suggesting limited postoperative predictive utility. Variables with substantial missing data (e.g., >30% missing C-reactive protein values) or multicollinearity (VIF >4.0 for fecal contamination) were excluded to ensure model stability [27]. Furthermore, our findings may not generalize to immunosuppressed populations, which were excluded from this study. These patients face unique diagnostic and management challenges, including elevated postoperative morbidity risks, warranting dedicated studies to develop tailored risk assessment tools [28].

Emerging biomarkers like serum butyrylcholinesterase show promise for enhancing risk stratification in colorectal surgery [29]. While not incorporated here, future research should evaluate their feasibility in low-resource settings, where cost and availability remain critical barriers. Integrating such biomarkers could refine predictions while maintaining practicality in constrained environments.

Risk score modeling, clinical implementation, and comparative analysis

In resource-limited settings lacking advanced diagnostics and minimally invasive surgery, simple risk scores based on routinely available clinical parameters are essential. These tools enable early identification of high-risk patients and targeted allocation of scarce resources, such as enhanced antibiotics, specialized wound care, and closer postoperative monitoring, thereby improving outcomes and reducing unnecessary interventions [6,25].

Our integer-based risk model for predicting SSIs after appendectomy demonstrated strong performance (AUC = 0.82) and effectively identified high-risk patients (positive predictive value = 82%). It relies on four accessible variables, i.e., appendiceal perforation, symptom duration >48 hours, ASA class ≥III, and operative time >60 minutes, ensuring applicability in low-resource environments. These findings align with established literature on SSI risk factors and offer several advantages for clinical implementation, as documented in supplementary materials (Supplementary Table) [6,13,30-35].

Compared to existing models, such as Emile et al.'s APSI [6] and the NoCtApp score [32], our model achieves similar predictive accuracy while excluding less reliably measured factors like BMI and diabetes, enhancing feasibility in settings with limited data availability. Although machine learning approaches report marginally higher accuracy, their complexity and data requirements limit their practical use in low-resource contexts [34,35]. Our model’s reliance on universally accessible clinical parameters offers a pragmatic balance between accuracy and applicability.

For high-risk patients (scores: 4-5; SSI rate: 52.4%), we recommend delayed wound closure, extended-spectrum antibiotics, and intensified surveillance. This targeted approach may reduce SSI-related morbidity and optimize resource use [30]. Future refinements could incorporate biochemical markers, where feasible, to further improve prediction.

Study limitations

This study has several inherent limitations. As a retrospective analysis, it is subject to residual confounding due to uncontrolled variations in surgical techniques and perioperative management. The relatively small number of SSIs (n = 34), particularly organ/space infections (n = 5), limited the statistical power for detailed subgroup analyses. Generalizability may be constrained by differences in patient demographics and institutional practices across LMICs. Additionally, as a multicenter study conducted mainly within academic institutions, the findings may not fully apply to community or private hospitals, where patient populations, surgical expertise, and resource availability differ.

The low utilization of laparoscopic appendectomy (<5%) precluded a comprehensive assessment of its potential protective effect against SSIs. Although the predictive model demonstrated satisfactory performance, external validation in populations with higher antimicrobial resistance prevalence is warranted. The absence of a cost-effectiveness analysis limits evaluation of whether risk-stratified interventions optimize resource allocation in constrained healthcare settings.

While this study reaffirms established SSI risk factors, such as appendiceal perforation and prolonged operative time, the integration of these variables into a validated, appendectomy-specific risk prediction model addresses a critical gap in resource-limited contexts where open appendectomy predominates and evidence-based tools are scarce. Future multicenter studies should refine this model by incorporating additional predictors, including surgical approach variables, and validate its applicability across diverse populations. Moreover, investigations into the economic impact of targeted prevention strategies and the integration of feasible biomarkers, such as procalcitonin and butyrylcholinesterase, may further enhance the model’s utility in low-resource environments.

## Conclusions

This study presents a clinically practical risk prediction model that effectively stratifies the risk of SSI following open appendectomy. Demonstrating robust predictive accuracy (AUC: 0.82) and reliable calibration, the model categorizes patients into three distinct risk groups with SSI probabilities ranging from 3.2% to 52.4%. By integrating four readily accessible clinical parameters, including appendiceal perforation, symptom duration, ASA classification, and operative time, the tool captures both disease severity and patient-specific vulnerability. Notably, it identifies a high-risk subgroup with a 22-fold increased likelihood of SSI, which may benefit most from targeted preventive measures.

The model’s strength lies in its methodological rigor, supported by bootstrap validation, and its practical applicability in routine clinical settings, particularly in resource-limited environments where open appendectomy remains predominant. Explaining 48% of the variance in SSI occurrence, the model offers meaningful predictive capacity while acknowledging opportunities for refinement through future research. Its straightforward scoring system addresses a critical gap in postoperative risk assessment, providing surgeons with an evidence-based instrument to guide infection prevention strategies. Future studies should focus on evaluating the model’s implementation in real-world clinical practice, emphasizing its potential to enhance antimicrobial stewardship and reduce SSI rates across diverse healthcare settings. Such investigations will be essential to determine how this risk stratification tool can optimally contribute to surgical quality improvement initiatives.

## Appendices

**Table 6:** Comparative analysis of risk prediction models for appendectomy complications in previous reports. AUC, area under the curve; OR, odds ratio; CI, confidence interval; ASA, American Society of Anesthesiologists; SSI, surgical site infection; CA, complicated appendicitis; LMIC, low- and middle-income country; APSI, appendicitis severity index; LightGBM, light gradient-boosting machine; DM, diabetes mellitus; PPV, positive predictive value; NPV, negative predictive value; CV, cross-validation; LASSO, least absolute shrinkage and selection operator. † Resource-intensive modality. Methodological footnotes: 1. All models employed internal validation (bootstrapping/cross-validation). 2. Calibration assessed via Hosmer-Lemeshow test, where reported (p > 0.05 = good fit). 3. † Marked models require specialized resources that may limit LMIC implementation.

Study (year)	Key predictors	Model characteristics	Performance metrics (95% CI)	Clinical utility	Implementation notes
Emile et al. (2021) [6]	Obesity (BMI >30), diabetes mellitus, free peritoneal fluid, perforated appendix	Multivariable logistic regression, internal validation	DM: OR = 6.05; perforation: OR = 24.64	SSI risk stratification	Requires BMI/DM screening
Feng et al. (2022) [30]	Pain >48 hours, peritonitis, bilirubin >1.2 mg/dL	Logistic regression, bootstrap validation	AUC: 0.985 (0.975-0.994)	Elderly CA prediction	Needs liver function tests
Noorit et al. (2018) [13]	Diabetes, incision >7 cm, fecal contamination, operative time >75 minutes	Logistic regression, bootstrapping	C-statistic: 0.74 (0.66-0.81)	Intra-operative risk assessment	Wound length measurement is critical
Zhao et al. (2024) [31]	Radiomics score, neutrophil %, fever >38°C	LASSO regression, radiomic analysis	AUC: 0.91 (vs. 0.85 CT alone)	Enhanced CT interpretation	†Requires radiomics expertise
Strohäker et al. (2023) [32]	Age, ASA ≥III, CRP >50 mg/L, symptoms >24 hours	NoCtApp score, multicenter validation	AUC: 0.861 (0.830-0.891)	Imaging-free CA rule-out	CRP availability needed
Avanesov et al. (2018) [33]	CT findings, age ≥52, fever, symptoms >48 hours	APSI score, radiologist review	PPV: 92%, NPV: 83%	CT-based CA prediction	†Requires CT availability
Wei et al. (2024) [34]	48 clinical/lab variables	Gradient boosting machine, 10-fold CV	Sensitivity: 91.7%, specificity: 97.4%	High-accuracy CA detection	†Computational resource-intensive
Yadalam et al. (2024) [35]	Periodontal indices, WBC, age	LightGBM algorithm	Accuracy: 98%	Comorbidity prediction	†Specialized dental assessment
Our study (2025)	Perforation, symptoms >48 hours, ASA ≥III, operative time >60 minutes	Multivariable regression, temperature scaling	AUC: 0.82 (0.76-0.88), Brier: 0.14	LMIC-adapted SSI prevention	Optimized for resource-limited settings
